# Condom use and non-use among transgender women in Colombia: a qualitative analysis based on the IMB model

**DOI:** 10.11606/s1518-8787.2022056004145

**Published:** 2022-09-16

**Authors:** Jorge Eduardo Moncayo Quevedo, María Del Mar Pérez-Arizabaleta, Wilmar Hernán Reyes Sevillano

**Affiliations:** I Universidad Antonio Nariño Facultad de Psicología Cali VAC Colombia Universidad Antonio Nariño. Facultad de Psicología. Cali, VAC, Colombia; II Universidad Cooperativa de Colombia Facultad de Psicología Cali VAC Colombia Universidad Cooperativa de Colombia. Facultad de Psicología. Cali, VAC, Colombia

**Keywords:** Women, Transgender Persons, Unsafe Sex, Sexual and Gender Minorities, psychology, Qualitative Research

## Abstract

**OBJECTIVE:**

Review the reasons for condom use and non-use among transgender women in Colombia based on the information, motivation and behavioral skills (IMB) model.

**METHOD:**

Qualitative study in which an iterative process analysis was carried out. A focal group participated in person, and in-depth interviews were conducted virtually.

**RESULTS:**

First study carried out in Colombia on condom use among transgender women under the IMB model. The information component finds that traditional sexual education does not have a positive impact. Regarding motivational aspects, the importance of family support and follow-up and community-based organizations to motivate sexual health care and condom use is highlighted. Regarding behavioral skills, it was found that distrust towards sexual partners and the acquisition of condoms promote their use.

**CONCLUSIONS:**

It is important to create spaces for sexual education delivered by and for the LGBTIQ population, followed by the medical knowledge of health centers, to have positive impacts on the sexual health of transgender women; studies with sexual partners of transgender women are encouraged in order to know the reasons why they request the non-use of condoms.

## INTRODUCTION

In recent years, studies on trans women (TW) have been increasing. The term *trans* in Colombia, and specifically for this study, refers to an umbrella concept that includes different gender expressions and identities, such as transsexual, transgender, transvestite, gender-neutral/fluid/gender queer people, etc. This category frames a vindictive political positioning, and includes gender identities that do not fit the binary model^1^. Literature shows that the most frequently researched topic with respect to this population has to do with the prevalence of the human immunodeficiency virus (HIV)^2^. There are some quantitative studies with trans women, HIV and the information, motivation and behavioral skills (IMB) model^6,7^. It relates to practices that may predispose to the infection from sexually transmitted infections (STIs). In relation to this discussion, the research team takes an ontological stance in which it distances itself from the relationship between TW and HIV declared under the concept of *key population*; in line with it, empirical data^8^ support the fact that, in Colombia, people living with the disease are not necessarily identified in any *key population*. Therefore, at the national level, the challenge is to focus health promotion and disease prevention strategies on the Colombian population in general.

In this context, condoms continue to be the best prevention tool^9,10^. However, their use by TW is not as consistent as expected^11,12^. This is due to several factors, both objective and subjective ones. The former has to do with material conditions, such as lack of money for access to condoms, and with external conditions, such as the reluctance of the partner to use them during sexual intercourse^13^. Subjective factors are associated with the so-called sexual education^14,15^, the history that is embodied and reproduced with respect to knowledge, beliefs, emotional state and the use of psychoactive substances, among others. Therefore, it is not possible to reduce the aforementioned practice to a rational choice tied to what is called the “logical acting”.

Given the importance and need for qualitative studies on condom use among TW and the relevance gained by the IMB model in studies to understand risk behaviors associated with STIs, especially HIV, and to inform the development of preventive interventions in different population groups with limited resources^16^, the present study aims to analyze the reasons for condom use and non-use among TW based on the IMB model.

The IMB model proposes that to the extent that people are informed and motivated, they engage in appropriate health care behaviors^19,20^. In this sense, this model includes three main constructs that influence behavioral changes: information, motivation and behavioral skills to perform the behavior^20,21^. *Information* refers to relevant knowledge about condoms, such as their role in preventing pregnancy and STIs. *Motivation* includes personal and social motivations; the former encompasses positive attitudes or benefits and negative attitudes toward condom use, while social motivation includes the individual’s perceptions of the social support provided by supportive networks available to promote condom use. *Behavioral skills* refer to the objective ability of individuals to have sex (oral, anal and/or vaginal) with a condom, and to the identification of factors that affect protected sexual practice by means of this method of protection^22^.

## METHOD

### Design

This is a qualitative study in which iterative process analysis was carried out^20,23,24^. In-depth focal group interviews were conducted, and discussed components of information, motivation and behavioral skills.

### Participants

The foundation that works with trans women called the participants chosen for the focal group (n = 11); the research group called them for the in-depth interviews (n = 22) by phone to confirm their participation, and if they met the conditions for making the interview and scheduling the meeting. It was ensured that participants had the necessary tools to carry out the focal group and interviews. Transportation allowance was offered (for the focal group) and internet connectivity was ensured, specifically to Google Meet for the interviews. The focal group lasted 180 minutes, and was conducted in January 2020. The in-depth interviews lasted 90–120 minutes, and were conducted from November 2020 to January 2021.

Participants (n = 33) were selected using the snowball strategy. Following were the eligibility criteria: trans women (transgender, transsexual, travesty, drag queen) of legal age (18 years old or more), residing in two of the main cities of the country (Cali and Medellín), and who voluntarily decided to participate in the study. For the eligibility of trans women, the two-question method was used, which was included in the sociodemographic data collected prior to the interviews and focal groups. All of them provided written and audio-recorded informed consent. Participants were paid $12 as transportation allowance and to ensure internet connectivity. The research team transcribed the interviews verbatim, and two team members reviewed these prior to data analysis.

### Focal Group and In-Depth Interviews

During January and December 2020, an in-person focal group and in-depth interviews were conducted virtually, due to the covid-19 health contingency.

The research team assembled both the focal group and the in-depth interview, which were reviewed by the foundation to ensure the greatest possible clarity in the use of language for the participants. This allowed for more in-depth information to enhance the analysis. The focal group had guidelines that encouraged participants to talk about information, motivation and behavioral skills on condom use through the dramatization of everyday scenes involving sexual health care, e.g., the relationship with the stable partner and with the client, contact with health centers and community-based organizations or foundations.

The in-depth interview guideline addressed the constructs of the IMB model. Information on condom use, on the motivations they have for taking care of their sexual health and, finally, on their sexual practices were asked regarding behavioral skills. At the end of each field (focal group and in-depth interviews), participants filled out a sociodemographic questionnaire that allowed the identification of contextual characteristics (Table 1).

**Table 1:** Sociodemographic characteristics of the participants (n = 33).

Variable	n (%)
Age (in years)	
20–24	2 (6.1)
25–29	6 (18.2)
30–34	3 (9.1)
35–39	5 (15.2)
40–44	5 (15.2)
45–49	3 (9.1)
≥ 50	9 (27.3)
Marital status	
Married	2 (6.1)
Polyamorous	1 (3.0)
Survivor	1 (3.0)
Unmarried	4 (12.1)
Single	24 (72.7)
Widowed	1 (3.0)
Race/Ethnicity	
African	8 (24.2)
Black	1 (3.0)
White	3 (9.1)
Indigenous	2 (6.1)
*Mestiza*	13 (39.4)
Mulatto	1 (3.0)
*Trigueña*	5 (15.2)
Occupation	
Alternate (stylist by day and sex worker by night)	4 (12.1)
Truck driver	1 (3.0)
Human rights advocate	7 (21.2)
Nurse	1 (3.0)
Stylist	7 (21.2)
Gardener	1 (3.0)
Webcam model	6 (18.2)
Various jobs	2 (6.1)
Sex worker	4 (12.1)
Monthly income (USD)	
0–50	8 (24.2)
51–100	4 (12.1)
100–180	3 (9.1)
181–260	4 (12.1)
261–385	6 (18.2)
385–515	2 (6.1)
≥ 516	6 (18.2)
Socioeconomic stratum	
1	8 (24.2)
2	8 (24.2)
3	15 (45.5)
5	2 (6.1)
Highest level of education attained	
Complete undergraduate degree	5 (15.2)
Incomplete undergraduate degree	5 (15.2)
Complete technician or vocational	7 (21.2)
Complete high school	9 (27.3)
Incomplete high school	4 (12.1)
Complete primary school	1 (3.0)
Incomplete primary school	1 (3.0)
None	1 (3.0)

### Information Analysis

Sociodemographic data were calculated by means of descriptive statistics (Table 1). Transcripts were entered into Nvivo software and coded by three members of the research group using the components of the IMB model. For the analysis, the iterative process^20,23,24^ was used to create a codebook based on the theoretical component of the IMB model. Subsequently, each researcher reviewed the coding to rectify and establish them under agreement of the whole team. The information from all the transcripts was organized in a matrix where a primary code and a secondary code were assigned to each component, to each of which an exemplary quote from the transcripts was assigned (Table 2). Coding inconsistencies were reviewed with the team until consensus was achieved.

**Table 2:** Recurring themes and exemplary quotes organized under the model of information, motivation and behavioral skills.

Theoretical component	Primary code	Secondary code	Exemplary quote
Information	Knowledge about condoms	Pregnancy prevention	The first time I heard about condoms was in school, and they related it to pregnancy prevention. At first my parents told me to use a condom so I wouldn’t get someone pregnant.
		STI prevention	When I started talking about HIV, I learned that it was a way to prevent HIV. I know that condoms protect against many diseases.
	Lack of knowledge	Lack of information	When I was young I didn’t know about condoms, I didn’t protect myself for a long time because I didn’t think it was necessary. When I began to have experience in sexual relations I learned about it. I had the habit of using double condoms and not using condoms. They have the idea that there is a risk in penetration but there is no risk in oral sex, so they protect themselves for penetration, but they do not protect themselves for oral sex.
Motivation	Personal motivational experiences	Self-care	One has to take care of the self, and value themselves as a human being, as a person. Over time I realized that I have to take care of my body, so I protect myself when I have sex. I always use it because I think it is a total danger not to use it, how scary to get a disease and damage your life, imagine the Aids, that you have to live with that, with constant treatments, how scary.
	Social motivational experiences	Acceptance by the blood and social family	When a trans girl counts on the support of her family, it greatly influences one’s desire to take care of one’s own health and that of others. In the community-based organization I go to (Santamaría Fundación) they motivate me to take care of my health, to use condoms and to make my partner respect me in my sexual practices. I am very careful in my sexual practices because I am afraid that my family will see me sick, and I will have to deal with an illness.
Behavior	Use	Mistrust of the sexual partner	I go with the client and at once I take out my condom and I uncover it and if the man has a problem because of the condom, I tell him that I am sorry but there is no service. If a man tells me that he pays me more for being without a condom, I already know that this is something dangerous and I refuse to have sex with him.
		Acquisition	I use it when I they are available. I can claim them at health centers and also at foundations. There are many organizations or foundations that give them away.
	Non-use	Characteristics of condoms	Condoms are too tight. Sometimes condoms cause allergies.
		Client’s conditions in sex work	There are trans women who are in need and it’s their turn, so they agree to sex without a condom when they are offered extra money. Nobody shoots porn with a condom anymore. One tries to put the condom on the toy, but if it appears a little bit the client makes them take it off.
		Relations with the sexual partner	The only thing that would influence the use of condoms is if the person is my steady partner for a long time, then it could be eliminated, but otherwise no. Men always say that they don’t feel with a condom. They get angry when you ask for it, and say it’s because they lose sensation. Besides, if you ask for it, they start distrusting you, saying that you are unfaithful.
Behavior	Non-use	Acquisition	There are very expensive condoms; people with limited economic resources cannot buy them because they have other needs. I was ashamed to go to a pharmacy to buy condoms.
		Disinhibition: attraction	When you like a man, the last thing you think about is using a condom. If you like the person, you can do anything in bed, even without a condom.
		Disinhibition: arousal	At the moment of arousal when you forget to use a condom, it doesn’t matter, you don’t even realize it.
		Disinhibition: consumption of psychoactive substances and alcohol	When I’m drunk I lose my mind, become irresponsible; everyone is irresponsible after being drunk. Alcohol makes us do many things, with a glass of alcohol you don’t care if it’s with or without a condom.
		Oral sex	Oral sex with a condom is horrible. I’m more flexible when it comes to oral sex. I do it without a condom.

STI: sexually transmitted infections; HIV: human immunodeficiency virus.

### Ethical Considerations

The research project was approved by the Research Ethics Committee of the Universidad Antonio Nariño. Each participant in the project expressed their informed consent prior to the focal group and in-depth interviews.

## RESULTS

### Characteristics of the Sample

The average age of participants was 41 years old. Of the total number of participants (n = 33), 51.5% (n = 17) have engaged in sex work at some point in their lives (6 virtually through webcam work, and 11 through street sex work); of these, 14 are currently engaged in sex work; the most frequent current occupations are styling (21.2%, n = 7) and human rights advocacy (21.2%, n = 7). Regarding the highest level of education attained, 21.2% (n = 7) did not complete basic education, 27.2% (n = 9) completed high school, 21.2% (n = 7) completed a technical or technological level, 15.1% (n = 5) have incomplete undergraduate degree, and 15.1% (n = 5) completed undergraduate degree. In terms of socioeconomic level, 93.4% (n = 31) are in strata 1, 2 and 3; in a disaggregated manner this corresponds to 24.2% (n = 8) in stratum 1, this same value for stratum 2, 45.4% (n = 15) in stratum 3, while only 6% (n = 2) are located in stratum 5 (see table of sociodemographic data). The sample characteristics are provided in Table 1.

### Recurrent Theme of the IMB Model: information.

**Knowledge about condoms:** All participants, at the time of the interviews, had information about condom. They knew that condoms are used to prevent pregnancy and sexually transmitted infections. The first information they received about condoms was at school and at their homes (families), and they referred to condoms as a pregnancy prevention method. Therefore, they associated condom use for cisgender women. Some participants associated condoms with HIV, gonorrhea, syphilis and papillomavirus. Most of the participants had information about where condoms are available, namely pharmacies, community-based organizations, and health centers. However, some of them did not buy condoms from these places because of embarrassment. All participants mentioned that as they experienced sexual life, they learned about STIs from their friends or sexual partners, which led them to learn more about condoms, and made condom use more important. The participants mentioned that the information on the use of condoms (how to store, open and use them) was acquired through their friends and some through community-based organizations that work to defend transgender people’s rights.

**Misinformation about condoms:** Most mentioned that at the time they started their first sexual relations they did not have information about condoms as a method of protection against STIs; for this reason, they had the erroneous idea that this method was only to prevent pregnancy, and should be used only by biological women. In addition, when they started having sex, they did not know the importance of using a condom for each penetration, and of opening the package carefully so as not to damage it. A quarter of the participants reported misunderstanding the use, thinking that by using two condoms at a single penetration they would be safer. Most respondents reported that at some point in their lives they were not clear about the use of condoms in oral sex, thinking it was not necessary to use them in this sexual practice. Participants were not sure about the different materials of condoms or the need for lubricants for their use in anal penetration.

### Recurrent Theme of the IMB Model: motivation.

**Motivational experiences:** These experiences were probed at the personal and social levels. At the personal level, awareness of self-care becomes the main motivation for using condoms, to avoid the consequences of an STI in one’s body. With regard to social motivation, the following experiences are found: acceptance, support and interest in health care by the family or supportive networks that motivate them to take care of their sexual health. Participants are motivated to care for themselves to avoid the physical consequences and the social stigma generated by STIs. All participants who have practiced sex work reported that clients motivate them not to use condoms in exchange for money. Trust in the relationship is a motivation for not using condoms with their regular sexual partners, according to a large number of participants.

### Recurrent Theme of the IMB Model: behavioral skills.

**Condom use:** Participants use condoms when they are suspicious of their sexual partner, i.e., when they suspect they may become infected with a disease. Some participants demand and use condoms in sex work, and this involves not receiving additional money. Most participants use condoms if they have them available at the time of sexual intercourse; they purchase them at health centers and, to a greater extent, at foundations or community-based organizations.

**Non-use of condoms:** The factors expressed by participants as affecting sexual practices with condoms are more abundant than those they refer with respect to use. Participants who had engaged in sex work reported not using condoms at some point in their work due to client’s request or demand. Most of the participants mentioned that it is common not to use condoms with their regular sexual partners. Participants in strata 1, 2 and 3 mentioned that sometimes they do not use condoms because of difficulties in purchasing them, either for economic reasons or because they are embarrassed to ask for them at a drugstore or health center. In addition to the above, all of the participants reported that they had not protected themselves during sexual intercourse at some point in their lives because they had had sex while consuming psychoactive substances and alcohol, as well as because of the excitement of the moment and the attraction they felt toward a casual sexual partner. A quarter of the participants reported not using condoms because of the characteristics of the condom, namely the size and material. Finally, all the participants mentioned that the sexual practice in which they were most likely not to use a condom was oral sex; they attributed this to the discomfort they and their sexual partners felt when using a condom.

## DISCUSSION

This study identified the reasons that TW have for condom use based on the IBM model. We identified external or contextual and internal elements, both relevant to the decision to use or not to use condoms. Despite the limited qualitative literature^23^ on this research topic, we found consistency with studies conducted in other contexts^25,26^ regarding the reasons for not using condoms. Results suggest challenges faced by TW in relation to contextual conditions of inequality that promote risky sexual practices.

Findings were consistent with quantitative studies on non-use of condoms among TW in different contexts^27^. It is common to find that trans women do not use condoms due to client demand in cases of sex work^29,32^, which exposes this risk. In the Colombian context^36,37^, as well as in other countries^38,39^, it is common for TW to have to engage in sex work due to the structural barriers they face when accessing formal employment. The results of this study also coincide with research conducted with people with heterosexual and trans gender identity in stating that condoms are not usually used with stable sexual partners^40^.

Other consistent findings are the recognition of the non-use of condoms due to disinhibition as a result of excitement^41^ and the consumption of psychoactive substances and liquor^42^. The practice of oral sex was a recurrent expression in this and previous studies^43^. It is common to find in literature that the first knowledge about condoms is related to its function of preventing pregnancy; studies suggest that this is because this method of protection is designed more for heterosexual couples^44^.

New findings were also observed in this study. The motivational experiences of acceptance and support to gender identity received from their blood and social families were related to motivation for self-care in general^45^; this was also related to the use of condoms as a protected sexual practice. We noticed that the information, which turned out to be significant for participants, on the use of condoms did not come directly from their blood families, but from their social families (trans friends who become a support network at an emotional, psychological and legal level). This study confirms the importance of the actions by community-based organizations, foundations and NGOs, in health care^28^. Similarly, these organizations promote the distribution of condoms, a factor that was mentioned in the behavioral skill to use condoms.

Although previous studies have found discrepancies between the relationship condom knowledge and practice, i.e., its use, some suggest that the relationship is effective^46^ and others suggest the opposite due to the lack of management and engagement of those who delivered the workshops on the topics^47^. This study proposes as challenge that pedagogical programs (which provide information) on condom use could have greater effect to the extent that they are delivered by the trans population that recognizes the experiences and life contexts of the TW. Additionally, participants were more open to the information delivered by community-based organizations, foundations, NGOs and social families, since these are spaces where they felt welcomed, cared for and defended through psychosocial accompaniment, in addition to being spaces of empowerment^48^. In this sense, information from these institutions is highly effective in the use of condoms.

Studies place TW as one of the main populations carrying STIs, especially HIV^32^. The results of this study show that one of the main reasons for non-use of condoms is due to the client’s request and demand in exchange for money. In Colombia, it is common for TW to engage in street sex work, due to the barriers they encounter in the educational and formal work sectors because of the stigma associated with their gender identity. It means that the sources of income generation are through informal work, including street sex work, in which they are exposed to risky sexual practices. This study shows the need to carry out studies with the sexual partners of TW, since it is found that they are the ones who promote, to a large extent, unprotected sexual practices^48,49^.

At the level of LGBTI public policy in Colombia^50^, there are no objectives specifically focused on the sexual health of transgender people or the living conditions that, as already mentioned, act as a risk factor for STIs. In this sense, it is recommended to include within these policies an axis that specifically addresses sexual health in which the distribution of condoms to trans women street sex workers is promoted, since the monthly income of the participants in this study does not allow them to cope with the monthly expenses with the condoms they use^51^.

In addition to the above, the findings of this study suggest strategies for disease prevention and sexual health promotion, specifically to promote increased condom use, thus reducing cases of STIs. Beyond promoting pedagogical spaces, it is essential that information about condoms and STIs be accompanied by the promotion of self-care. Likewise, the information should be provided by people they trust in, and who do not judge their gender identity, sexual orientation, and sexual practices.

### Limitations

This study has some limitations: the sample size does not allow generalizations, as it was based on convenience and intentionality, as well as voluntary participation and snowball strategy, something that occurs very frequently in studies with transgender population, as shown in studies^34,52,53^. Therefore, the sample of this study presents a high degree of homogeneity, which in turn is related to the difficulties of access to the population studied.

The snowball technique led us to recruit subjects from strata 1, 2 and 3 with incomes below the legal minimum wage in force in Colombia (approximately 280 dollars). This has limited the possibility of analyzing the wealthier population. We also recognize that there are other socio-cultural discourses and knowledge relevant to understanding the phenomenon studied, which did not emerge in our interviews.

## CONCLUSION

Although the term “key population” assigned to specific populations, including trans women, is currently being debated and analyzed in Colombia^8^, studies are needed to understand the sexual practices demanded by trans women’s sexual partners (clients or steady partners), as well as social and political efforts to improve the possibilities of access to formal work for trans women in Colombia. The findings of this study provide an important basis for the development of future interventions to promote condom use among trans women. For the positive impact of these interventions, strategies that are adapted to the population become indispensable, e.g., that leadership is carried out by the trans population that recognizes the dynamics and life experiences of participants.
