# Supplementary material for: Condom use and non-use among transgender women in Colombia: a qualitative analysis based on the IMB model
Source: Rev Saude Publica. 2022 Sep 16;56:84. doi: 10.11606/s1518-8787.2022056004145 (PMC9529206; doi:10.11606/s1518-8787.2022056004145)
Supplement: Translation

# Translation: El uso y no uso del condón en mujeres trans de Colombia: un análisis cualitativo desde el modelo IMB

**Authors:** Jorge Eduardo Moncayo Quevedo, María Del Mar Pérez-Arizabaleta, Wilmar Hernán Reyes Sevillano

**Affiliations:** I Colombia Universidad Antonio Nariño. Facultad de Psicología. Cali, VAC, Colombia; II Colombia Universidad Cooperativa de Colombia. Facultad de Psicología. Cali, VAC, Colombia

**Keywords:** Mujeres, Personas Transgénero, Sexo sin proteción, Minorías Sexuales y de Género, psicología, Investigación Cualitativa

## Abstract

**OBJETIVO:**

Analizar las razones del uso y no uso del condón que tienen las mujeres trans de Colombia desde el modelo de información, motivación y habilidades conductuales (IMB por sus siglas en inglés).

**MÉTODO:**

Estudio cualitativo en el cual se llevó a cabo el análisis de proceso iterativo. Se realizó un grupo focal de manera presencial y entrevistas a profundidad de manera virtual.

**RESULTADOS:**

Primer estudio llevado a cabo en Colombia sobre el uso del preservativo en mujeres trans bajo el modelo IMB. En el componente información se identifica que la educación sexual tradicional no tiene un impacto positivo. Respecto a lo motivacional, se destaca la importancia del apoyo y acompañamiento familiar y de las organizaciones de base comunitaria para motivar al cuidado de la salud sexual y el uso del preservativo. Con relación a las habilidades conductuales, se analiza que la desconfianza hacia la pareja sexual y la adquisición de los preservativos promueven su uso.

**CONCLUSIONES:**

Se hace importante la creación de espacios de educación sexual realizados por y para la población LGBTIQ, acompañados por el saber médico de los centros de salud, para impactar de manera positiva la salud sexual de mujeres trans; se motiva a la realización de estudios con parejas sexuales de mujeres trans para conocer las razones por las cuales solicitan el no uso del preservativo.

## INTRODUCCIÓN

En los últimos años los estudios sobre mujeres trans (MT) han ido en aumento. El término *trans,* en Colombia, y específicamente para este estudio, hace referencia a un concepto sombrilla que incluye diferentes expresiones e identidades de género, tales como transexual, transgénero, travesti, personas con género neutro/fluido/ *gender queer*, etc. Esta categoría enmarca un posicionamiento político reivindicativo e incluye identidades de género que se encuentran al margen del modelo binario^1^. La literatura da cuenta de que el tema que se investiga con mayor frecuencia respecto a esta población tiene que ver con la prevalencia del virus de inmunodeficiencia humana (VIH)^2^. Hay algunos estudios cuantitativos con mujeres trans, el VIH y el modelo de información, motivación y habilidades conductuales (IMB por sus siglas en inglés – *information, motivation and behaviour*)^6,7^. Esto se relaciona a prácticas que pueden predisponer al contagio de infecciones de transmisión sexual (ITS). A propósito de esta discusión, el equipo de investigación marca un posicionamiento ontológico en el cual se distancia de la relación entre MT y VIH declarada bajo el concepto de *población clave*; en coherencia con esto, datos empíricos^8^ permiten soportar que, en Colombia, las personas que viven con la enfermedad no se identifican necesariamente en ninguna *población clave*, por lo tanto a nivel nacional la apuesta está en dirigir estrategias de promoción de la salud y prevención de la enfermedad a la población colombiana en general.

En este marco, el condón sigue siendo aún la mejor herramienta de prevención^9,10^; no obstante, su uso por parte de las MT no presenta la consistencia esperada^11,12^. Esto obedece a varios factores, ya sean objetivos o subjetivos. Los primeros tienen que ver con condiciones materiales, como la falta de dinero para el acceso al preservativo, y con condiciones externas, como la renuencia de la pareja a usarlo durante en el encuentro sexual^13^. Los factores subjetivos se asocian con la denominada educación sexual^14,15^, lo historizado que se encarna y reproduce respecto al conocimiento, a las creencias, al estado emocional y al uso de sustancias psicoactivas, entre otros; por ello no es posible reducir la mencionada práctica a una elección racional anudada a lo que se llama el “lógico actuar”.

Debido a la importancia y necesidad de estudios cualitativos sobre el uso del preservativo en MT y a la relevancia que ha cobrado el modelo IMB en los estudios para comprender los comportamientos de riesgo asociados con las ITS, en especial el VIH, y para informar sobre el desarrollo de intervenciones preventivas en diferentes grupos poblacionales con recursos limitados^16^, el presente estudio tiene como objetivo analizar las razones del uso y no uso del condón que tienen las MT desde el modelo IMB.

El modelo IMB propone que en la medida que las personas están informadas y motivadas, se involucran en conductas adecuadas del cuidado de la salud^19,20^. En este sentido, este modelo incluye tres constructos principales que influyen en los cambios de comportamiento: la información, la motivación y las habilidades conductuales para realizar el comportamiento^20,21^. Por *información* se entenderá el conocimiento relevante sobre el preservativo, como su función de prevención de embarazos y de ITS. La *motivación* incluye motivaciones a nivel personal y social; la primera de estas abarca las actitudes positivas o beneficios y negativas hacia el uso del preservativo, y la motivación social incluye las percepciones que tiene el individuo del apoyo social de las redes de apoyo con las que cuenta para promover el uso del preservativo. Las *habilidades conductuales* hacen referencia a la capacidad objetivo de las personas para tener relaciones sexuales (orales, anales y/o vaginales) con preservativo y a la identificación de factores que afectan la práctica sexual protegida por medio de este método de protección^22^.

## MÉTODO

### Diseño

Este es un estudio cualitativo en el cual se llevó a cabo el análisis de proceso iterativo^20,23,24^. Se realizaron entrevistas a profundidad en un grupo focal en las cuales se discutieron los componentes de información, motivación y habilidades conductuales respecto al uso del preservativo.

### Participantes

La fundación que trabaja con mujeres trans convocó a las participantes elegidas para el grupo focal (n = 11); el grupo de investigación las llamó para las entrevistas a profundidad (n = 22), vía telefónica para que confirmaran la participación y cumplieran las condiciones para realizar la entrevista y programar el encuentro. Se aseguró que las participantes contaran con las herramientas necesarias para la realización del grupo focal y de las entrevistas. Se ofreció un auxilio de transporte (para el grupo focal) y se aseguró la conectividad a internet, específicamente a Google Meet para el caso de las entrevistas. El grupo focal tuvo una duración de 180 minutos y fue llevado a cabo en enero de 2020. Las entrevistas a profundidad tuvieron una duración de 90 a 120 minutos y se realizaron entre noviembre de 2020 y enero de 2021.

Las participantes (n = 33) fueron seleccionadas utilizando la estrategia de bola de nieve, y los criterios de elegibilidad fueron: mujeres trans (transgénero, transexual, travesti, transformista) mayores de edad (18 años), que residan en dos de las principales ciudades del país (Cali y Medellín) y que decidan participar de manera voluntaria en el estudio. Para la elegibilidad de mujeres trans, se usó el método de las dos preguntas, las cuales estaban incluidas en los datos sociodemográficos recogidos previo a las entrevistas y grupos focales. Todas proporcionaron consentimiento informado por escrito y por grabación de audio. Las participantes recibieron 12 USD por concepto de auxilio de transporte y para asegurar la conectividad a internet. El equipo de investigación transcribió textualmente las entrevistas y dos miembros del equipo las revisó previo al análisis de la información.

### El Grupo Focal y la Entrevista a Profundidad

Durante enero y diciembre de 2020 se realizó un grupo focal de manera presencial y entrevistas a profundidad de manera virtual, debido a la contingencia sanitaria por el covid-19.

El equipo de investigación construyó tanto el grupo focal como la entrevista a profundidad los que fueron revisados por la fundación con el objetivo de contar con la mayor claridad posible en el uso del lenguaje para las participantes, esto permitió profundizar información para potencializar el análisis. El grupo focal contó con una guía donde se motivó a las participantes a hablar sobre la información, la motivación y las habilidades conductuales sobre el uso del preservativo por medio de la dramatización de escenas cotidianas que involucren el cuidado de la salud sexual, por ejemplo, la relación con la pareja estable y con el cliente, el contacto con centros de salud y organizaciones de base comunitaria o fundaciones.

La guía de la entrevista a profundidad abordó los constructos del modelo IMB. Se indagaron las informaciones del preservativo, sobre las motivaciones que tienen para cuidar de su salud sexual y, por último, sobre sus prácticas sexuales para indagar las habilidades conductuales. Al finalizar cada uno de los espacios (grupo focal y entrevistas a profundidad), las participantes diligenciaron un cuestionario sociodemográfico que permitió conocer características contextuales (Tabla 1).

**Tabla 1:** Características sociodemográficas de las participantes (n = 33).

Variable	n (%)
Edad (en años)	
20–24	2 (6,1)
25–29	6 (18,2)
30–34	3 (9,1)
35–39	5 (15,2)
40–44	5 (15,2)
45–49	3 (9,1)
≥ 50	9 (27,3)
Estado civil	
Casada	2 (6,1)
Poliamor	1 (3,0)
Sobreviviente	1 (3,0)
Unión libre	4 (12,1)
Soltera	24 (72,7)
Viuda	1 (3,0)
Raza/etnia	
Afro	8 (24,2)
Negra	1 (3,0)
Blanca	3 (9,1)
Indígena	2 (6,1)
Mestiza	13 (39,4)
Mulata	1 (3,0)
Trigueña	5 (15,2)
Ocupación	
Alternante (estilista de día y trabajadora sexual de noche)	4 (12,1)
Conductora de camión	1 (3,0)
Defensora de derechos humanos	7 (21,2)
Enfermera	1 (3,0)
Estilista	7 (21,2)
Jardinera	1 (3,0)
Modelo webcam	6 (18,2)
Oficios varios	2 (6,1)
Trabajadora sexual	4 (12,1)
Ingresos mensuales (USD)	
0–50	8 (24,2)
51–100	4 (12,1)
100–180	3 (9,1)
181–260	4 (12,1)
261–385	6 (18,2)
385–515	2 (6,1)
≥ 516	6 (18,2)
Estrato socioeconómico	
1	8 (24,2)
2	8 (24,2)
3	15 (45,5)
5	2 (6,1)
Máximo nivel educativo alcanzado	
Pregrado completo	5 (15,2)
Pregrado incompleto	5 (15,2)
Técnico o tecnológico completo	7 (21,2)
Bachillerato completo	9 (27,3)
Bachillerato incompleto	4 (12,1)
Primaria completa	1 (3,0)
Primaria incompleta	1 (3,0)
Ninguno	1 (3,0)

### Análisis de la Información

Los datos sociodemográficos se calcularon por medio de estadística descriptiva (Tabla 1). Las transcripciones se incorporación al software Nvivo y fueron codificadas por tres miembros del grupo de investigación utilizando los componentes del modelo IMB. Para el análisis se empleó el proceso iterativo^20,23,24^ al crear un libro de códigos, teniendo como base el componente teórico del modelo IMB. Posterior a esto, cada investigador revisó las codificaciones para rectificarlos y establecerlos bajo acuerdo de todo el equipo. Se organizó la información de todas las transcripciones en una matriz donde se asignó un código primario y un código secundario por componente, a cada uno de estos se asignó una cita ejemplar de las transcripciones (Tabla 2). Las inconsistencias de codificación se revisaron con el equipo hasta el punto de llegar a acuerdos.

**Tabla 2:** Temas recurrentes y citas ejemplares organizadas bajo el modelo de información, motivación y habilidades conductuales.

Componente teórico	Código primario	Código secundario	Cita ejemplar
Información	Conocimiento sobre el preservativo	Prevención de embarazos	La primera vez que escuché hablar del condón fue en la escuela y lo relacionaban con la prevención de embarazos. A principio me decían mis papás que usara condón para no dejar a alguien embarazada.
		Prevención de ITS	Cuando empieza con el tema del VIH empecé a conocer que era una forma de prevenir el VIH. Del preservativo conozco que sirve para proteger de muchas enfermedades.
	Desconocimiento	Desinformación	Cuando era joven yo no conocía el condón, yo mucho tiempo no me protegí, porque yo no lo veía necesario. Cuando empecé a tener experiencia en las relaciones sexuales es que lo conocí. Yo tenía la costumbre de poner doble condón y de no usar preservativo. Se tiene la idea que en la penetración si hay riesgo pero que en el sexo oral no hay riesgo, entonces se protegen para la penetración, pero no se protegen para el sexo oral.
Motivación	Experiencias motivadoras personales	El autocuidado	Uno tiene que tener autocuidado y valorarse como ser humano, como persona. Con el tiempo yo me di cuenta que mi cuerpo lo tengo que cuidar, entonces por eso me protejo cuando tengo relaciones. Siempre lo uso porque pienso que es un peligro total no usarlo, qué miedo una enfermedad y dañarse la vida, pero dígame un sida, que usted tiene que vivir con eso, con tratamientos constantemente, qué miedo.
	Experiencias motivadoras sociales	Aceptación de la familia consanguínea y social	Cuando una chica trans cuenta con el apoyo de su familia influye mucho en el deseo que uno tenga de cuidarse la propia salud y la de los demás. En la organización de base comunitaria a la que voy (Santamaría Fundación) me motivan a cuidar de mi salud, a usar el condón y hacerme respetar en las prácticas sexuales. Yo me cuido mucho en las prácticas sexuales porque da miedo que mi familia me tenga que ver enferma y tenga que lidiarme una enfermedad.
Comportamiento	El uso	Desconfianza de la pareja sexual	Yo voy con el cliente y de una vez saco mi preservativo y ya ahí lo destapo y si el hombre pone problema por el preservativo, pues le digo que lo siento pero que no hay servicio. Si un hombre me dice que me paga más por estar sin preservativo yo ya sé que eso es algo peligroso y me rehúso a tener relaciones con él.
		La adquisición	Lo utilizo cuando los tengo a disposición. En los centros de salud los puedo reclamar y también en fundaciones. Hay muchas organizaciones o fundaciones que los regalan.
	El no uso	Características del preservativo	El preservativo aprieta demasiado. En ocasiones el preservativo causa alergia.
		Las condiciones del cliente en el trabajo sexual	Hay chicas que tienen muchas necesidades y les toca, entonces acceden al sexo sin condón cuando les ofrecen dinero de más. Ya nadie graba porno con condón. Uno intenta colocar el preservativo al juguete, pero si se ve un poco el cliente lo hace quitar.
		Relaciones con la pareja sexual	Lo único que influiría pues en el uso del condón es que sea mi pareja estable de mucho tiempo ya pues se podría eliminar, pero de resto no. Los hombres siempre dicen que no sienten con preservativo. Se enojan cuando uno lo pide y dicen que es porque pierden la sensación. Además, si uno lo pide empiezan a desconfiar, a decir que una es infiel.
Comportamiento	El no uso	La adquisición	Hay preservativos muy costosos, la gente de escasos recursos económicos no puede comprarlos porque tienen otras necesidades. Me daba pena ir a una droguería por condones.
		La desinhibición: la atracción	Cuando a uno le gusta el hombre, pues lo último que piensa uno es en utilizar un condón. Si a uno le gusta la persona, en la cama se vale lo que sea, hasta hacerlo sin preservativo.
		La desinhibición: la excitación	En el momento como de la excitación que a uno se le olvida el condón, como que no le importa, o sea uno no cae ni en cuenta de eso.
		La desinhibición: el consumo de sustancias psicoactivas y alcohol	Estando borracha pierdo la noción ya, uno es irresponsable; todo el mundo después de estar borracho es irresponsable. El alcohol lleva muchas cosas, con una copa de alcohol ya uno no le importa si con o sin preservativo.
		El sexo oral	El sexo oral con condón es muy horrible. En el oral soy más flexible. Lo hago sin preservativo.

ITS: infecciones de transmisión sexual; VIH: virus de inmunodeficiencia humana.

### Consideraciones Éticas

El proyecto de investigación fue aprobado por el Comité de Ética en la Investigación de la Universidad Antonio Nariño. Cada participante del proyecto expresó el consentimiento informado antes del grupo focal y de las entrevistas a profundidad.

## RESULTADOS

### Características de la Muestra

El promedio de edad de las participantes es de 41 años. Del total de las participantes (n = 33), el 51,5% (n = 17) han ejercido el trabajo sexual en algún momento de sus vidas (6 de manera virtual por medio del ejercicio webcam y 11 por medio del trabajo sexual de calle); de estas, en la actualidad lo ejercen 14; las ocupaciones actuales más frecuentes son el estilismo (21,2%, n = 7) y la defensa de derechos humanos (21,2%, n = 7). Con relación al máximo nivel educativo alcanzado, el 21,2% (n = 7) no finalizó la educación básica, el 27,2% (n = 9) concluyó el bachillerato, el 21,2% (n = 7) completó el nivel técnico o tecnológico, el 15,1% (n = 5) tiene un pregrado incompleto y el 15,1% (n = 5) culminó el pregrado. En cuanto al nivel socioeconómico, el 93,4% (n = 31) se encuentra en los estratos 1, 2 y 3, de manera desagregada esto corresponde al 24,2% (n = 8) en estrato 1, este mismo valor para el estrato 2, el 45,4% (n = 15) en el estrato 3, mientras que tan solo el 6% (n = 2) se ubica en el estrato 5 (verla tabla de datos sociodemográficos). Las características de la muestra se proporcionan en la Tabla 1.

### Tema Recurrente del Modelo IMB: información

**Conocimiento del preservativo:** Todas las participantes, al momento de realizar las entrevistas, tenían información sobre el preservativo. Sabían que el preservativo sirve para la prevención de embarazos y de infecciones de transmisión sexual. Las primeras informaciones que recibieron sobre el preservativo fueron en las escuelas y en sus hogares (familias) y referían al preservativo como método de prevención de embarazos, por lo tanto, relacionaban el uso del preservativo para mujeres cisgénero. Algunas participantes asociaron el preservativo con VIH, gonorrea, sífilis y papiloma. La mayoría de las participantes tenía informaciones sobre los lugares donde se pueden conseguir los preservativos, a saber, farmacias, organizaciones de base comunitaria y centros de salud; no obstante, algunas de ellas no lo compraban en estos lugares por vergüenza. Todas las participantes mencionaron que en la medida que experimentaban la vida sexual conocieron, por medio de sus amistades o parejas sexuales, sobre la existencia de las ITS; con esto profundizaron en la información del preservativo y cobró relevancia su uso. Las participantes mencionaron que la información sobre el uso (manera de almacenarlos, de abrirlos y utilizarlos) la adquirieron por sus amistades y algunas por medio de organizaciones de base comunitaria que trabajan en la reivindicación de derechos de personas trans.

**La desinformación sobre el preservativo:** La mayoría mencionó que en la época en que iniciaron sus primeras relaciones sexuales no contaron con informaciones sobre el preservativo como método de protección de las ITS; por esta razón, tenían la idea errónea de que este método solo era para prevenir el embarazo, por lo tanto, lo debían usar únicamente mujeres biológicas. Sumado a esto, cuando iniciaron la vida sexual, no sabían de la importancia de utilizar un preservativo por cada penetración y de abrir el empaque con cuidado para no dañarlo. Una cuarta parte de las participantes relató haber tenido un malentendido sobre el uso, pensando que al utilizar dos preservativos en una sola penetración iban a estar más seguras. La mayoría refirió que en algún momento de su vida no tuvieron claridad sobre el uso del preservativo en el sexo oral, pues pensaban que no era necesario utilizarlo en esta práctica sexual. No había seguridad de las participantes sobre los diferentes materiales de los preservativos ni de la necesidad de lubricantes para su uso en la penetración anal.

### Tema Recurrente del Modelo IMB: motivación

**Experiencias motivadoras:** Estas experiencias se indagaron a nivel personal y social. Con relación al nivel personal, la consciencia del autocuidado se convierte en la principal motivación de utilizar el preservativo, esto por el hecho de evitar las secuelas de una ITS en el propio cuerpo. Con relación a la motivación social, se encuentran las siguientes experiencias: aceptación, apoyo e interés del cuidado de la salud que tiene la familia o redes de apoyo sobre ellas que las motiva a cuidar de la salud sexual. Las participantes se motivan al cuidado por evitar las secuelas físicas y por el estigma social que generan las ITS. La totalidad de participantes que han ejercido el trabajo sexual han referido que los clientes las motivan a no utilizar el preservativo a cambio de dinero. La confianza en la relación es una motivación para no utilizar el preservativo con sus parejas sexuales estables según indica gran parte de las participantes.

### Tema Recurrente del Modelo IMB: habilidades conductuales

**El uso del preservativo:** Las participantes utilizan el preservativo cuando desconfían de su pareja sexual, es decir, cuando sospechan que pueden infectarse de una enfermedad. Algunas participantes exigen y utilizan el preservativo en el trabajo sexual y esto implica no recibir dinero adicional. La mayoría de las participantes utilizan el preservativo si lo tienen a disposición en el momento de la relación sexual, estos los adquieren en centros de salud y, en mayor cantidad, en fundaciones u organizaciones de base comunitaria.

**El no uso del preservativo:** Los factores manifestados por las participantes que afectan las prácticas sexuales con preservativo son más abundantes que los que refieren respecto al uso. Las participantes que habían ejercido el trabajo sexual refirieron no haberlo utilizado en algún momento del ejercicio de su trabajo debido a la solicitud o exigencia del cliente. La mayoría de las participantes mencionó que es frecuente no utilizar el preservativo con sus parejas sexuales estables. Las participantes de estratos 1, 2 y 3 mencionaron que en ocasiones no utilizan el preservativo por dificultades en la compra, ya sea por razones económicas o por vergüenza de solicitarlo en una droguería o centro de salud. Sumado a lo anterior, la totalidad de las participantes refirió no haberse protegido en la relación sexual en algún momento de sus vidas por haber tenido relaciones sexuales en medio del consumo de sustancias psicoactivas y alcohol, como también por la excitación del momento y la atracción sentida hacia la pareja sexual casual. Una cuarta parte relató no utilizarlo por las características del preservativo, a saber el tamaño y el material. Para finalizar, todas las participantes mencionaron que la práctica sexual en la cual hay más probabilidades de no usar el preservativo es en el sexo oral; esto lo atribuyen a la incomodidad que les genera a ellas y a sus parejas sexuales realizarla con preservativo.

## DISCUSIÓN

Este estudio identificó las razones que tienen las MT respecto al uso del preservativo desde el modelo IMB. Identificamos elementos externos o contextuales e internos, ambos relevantes para la toma de decisión del uso y del no uso del preservativo. A pesar de la poca literatura respecto a este tema de investigación de manera cualitativa^23^, en lo relacionado con las razones sobre el no uso del condón, se encuentra consistencia con estudios realizados en otros contextos^25,26^. Lo planteado en los resultados sugiere desafíos que enfrentan las MT a propósito de condiciones contextuales de desigualdad que promueve prácticas sexuales de riesgo.

Los hallazgos fueron consistentes con estudios cuantitativos sobre el no uso del condón en MT en diferentes contextos^27^. Es común encontrar que las mujeres trans no utilizan el preservativo por exigencia del cliente en los casos de trabajo sexual^29,32^, lo que expone este riesgo. En el contexto colombiano^36,37^, como también en otros países^38,39^, es frecuente que las MT deban ejercer el trabajo sexual a causa de las barreras estructurales que tienen al momento de acceder a un trabajo formal. Los resultados de este estudio también coinciden con investigaciones realizadas con personas con identidad de género heterosexual y trans al plantear que con parejas sexuales estables no suele utilizarse el preservativo^40^.

Otros hallazgos consistentes radican en el reconocimiento del no uso del preservativo por la desinhibición a causa de la excitación^41^ y el consumo de sustancias psicoactivas y licor^42^. La práctica del sexo oral fue una expresión recurrente en este estudio y en previos^43^. Es frecuente encontrar en la literatura que los primeros conocimientos sobre el preservativo están relacionados con su función de prevención del embarazo; los estudios plantean que esto se debe a que este método de protección está pensado en mayor proporción para parejas heterosexuales^44^.

En este estudio también se observaron hallazgos nuevos. Las experiencias motivadoras de aceptación y apoyo de la identidad de género recibido de sus familias consanguíneas y sociales se relacionan con la motivación al autocuidado de la salud –en general–^45^; esto mismo se relacionó con el uso del preservativo como práctica sexual protegida. Notamos que la información, que resultó ser significativa para las participantes, sobre el uso del preservativo no provenía directamente de las familias consanguíneas, sino de sus familias sociales (amigas trans que se convierten en red de apoyo a nivel emocional, psicológico y jurídico); este estudio ratifica la importancia de las acciones de las organizaciones de base comunitaria, las fundaciones y ONG, en el cuidado de la salud^28^. De igual manera, estas organizaciones promueven la distribución de los preservativos, un factor que se mencionó en la habilidad conductual del uso.

Aunque estudios previos han encontrado discrepancias entre la relación conocimiento del preservativo y la práctica, es decir, su uso, unos plantean que la relación es efectiva^46^ y otros proponen lo contrario debido al poco manejo e implicación que tenían quienes dictaban los talleres sobre los temas^47^; este estudio plantea como hallazgo que los programas pedagógicos (que proporcionan información) sobre el uso del preservativo podrían tener mayor efecto en la medida que sean dictados por la población trans que reconozca las experiencias y contextos de vida de las MT. Adicionalmente, las participantes fueron más receptivas a las informaciones provenientes de las organizaciones de base comunitaria, fundaciones, ONG y familias sociales, ya que son espacios en los que se sintieron acogidas, cuidadas y defendidas por medio del acompañamiento psicosocial, además de ser espacios de empoderamiento^48^. En este sentido, la información proveniente de estas instituciones tiene una alta efectividad en el uso del preservativo.

Estudios ubican a las MT como una de las principales poblaciones portadoras del ITS, en especial de VIH^32^. En los resultados de este estudio se expone que una de las principales razones del no uso del preservativo se debe a la solicitud y exigencia del cliente a cambio de dinero. En Colombia, es común que las MT se dediquen al trabajo sexual de calle, debido a las barreras que encuentran en el sector educativo y del trabajo formal por el estigma asociado a su identidad de género; esto hace que las fuentes de generación de ingresos económicos sean por medio del trabajo informal, dentro de esos el trabajo sexual de calle, en el que están expuestas a prácticas sexuales de riesgo. Este estudio muestra la necesidad de llevar a cabo estudios con las parejas sexuales de las MT, ya que se encuentra que son quienes promueven, en gran medida, las prácticas sexuales sin protección^48,49^.

A nivel de política pública LGBTI en Colombia^50^, no se identifican objetivos centrados específicamente a la salud sexual de las personas trans ni a las condiciones de vida que, como ya se mencionó, actúan como factor de riesgo para las ITS. En este sentido, se recomienda incluir dentro de estas políticas un eje que aborde, en específico, la salud sexual en el que se promueva la distribución de preservativos a mujeres trans trabajadoras sexuales de calle, ya que los ingresos mensuales de las participantes de este estudio no permiten cubrir los gastos en preservativos mensuales que utilizan^51^.

Sumado a lo anterior, los hallazgos de este estudio sugieren estrategias de prevención de la enfermedad y promoción de la salud sexual; en específico para promover el aumento en el uso del preservativo y con esto disminuir los casos de ITS. Más allá de promover espacios pedagógicos, es esencial que la información sobre el preservativo y las ITS esté acompañada de la promoción del autocuidado y que la información sea suministrada por personas con quienes tengan confianza y que no juzguen su identidad de género, orientación sexual y prácticas sexuales.

### Limitaciones

Este estudio cuenta con algunas limitaciones: el tamaño de la muestra no permite hacer generalizaciones; pues esta fue por conveniencia e intencional, además por participación voluntaria y por estrategia bola de nieve, algo que ocurre con mucha frecuencia en estudios con población trans, tal como lo muestran los estudios^34,52,53^. Por lo anterior, la muestra de este trabajo presenta un alto grado de homogeneidad, lo que a su vez se relaciona con las dificultades de acceso a la población estudiada.

La técnica de bola nieve nos condujo en mayor medida a reclutar sujetos de estratos 1, 2 y 3 con ingresos económicos por debajo del salario mínimo legal vigente en Colombia (280 dólares aproximadamente); esto limitó la posibilidad de atender el análisis en población con más recursos económicos. Reconocemos también que hay otros discursos y saberes de orden sociocultural relevantes para comprender el fenómeno estudiado, que no han surgido en nuestras entrevistas.

## CONCLUSIÓN

Aunque en la actualidad el término de “población clave” que se atribuye a poblaciones específicas, entre esas las mujeres trans, está siendo debatido y analizado en Colombia^8^, se requieren estudios que permitan conocer las prácticas sexuales que exigen las parejas sexuales (clientes o parejas estables) de las mujeres trans, así como los esfuerzos sociales y políticos para mejorar las posibilidades de acceso al trabajo formal de estas en Colombia. Los hallazgos de este estudio proporcionan una base importante para el desarrollo de futuras intervenciones que promuevan el uso del preservativo entre mujeres trans. Para el impacto positivo de estas intervenciones se hacen indispensables estrategias que se adapten a la población, por ejemplo, que el liderazgo sea llevado a cabo por la población trans que reconozca las dinámicas y experiencias de vida de las participantes.
